# 

**Published:** 1924-04

**Authors:** 


### HOSPITAL and HEALTH REVIEW

*Established 1886* *No. 1873. Vol. LXXIII*

*New Series.* Vol. III. No. 31. *April,* 1924. Price Sixpence

CONTENTS.

| | PAGE |
|---|---|
| Defects of the Lunacy Law | 97 |
| Rural Housing and Rural Sickness | 98 |
| Notes and Comments:—What Causes Rheumatism?—The Traffic in Narcotics—Town and Suburban Hospitals—A Partisan Rent Bill—Decadent Man—The Cost of Street Collections—Dangers of Artificial Malaria—"Dragons'-Teeth" as Cures—The Health Play—Old at Sixty!—Everything but Food—The Elimination of Diphtheria—Dental Clinics—Health of Chicago Children | 99 |
| Hospital Men of Mark: Sir Edward Pearson and Mr. C. M. Power | 103 |
| The Conditions of Health: Can We Secure Them? | 104 |
| Qualifications of a Hospital Secretary | 105 |
| Red Cross Hospital Services: Dr. Kay Menzies as Administrator | 106 |
| The Care of the Baby | 106 |
| Hospital Progress and Finance | 107 |
| What Manner of Man is the Murderer? | 109 |
| A Mission Hospital in Assam | 110 |
| Health in the Irish Free State | 110 |
| The Public Health: Interviews with Local Authorities: Bristol | 112 |
| The Convalescent Mental Patient | 114 |
| Correspondence | 115 |
| The Elizabeth Garrett-Anderson Hospital | 116 |
| Hospital Officers' Association | 116 |
| Reviews of Books | 117 |
| Echoes of the Month | 121 |
| Hospital and Health News | 122 |
| Points from Hospital Reports | 124 |
| Public Health in Parliament | 125 |
| Recent Appointments | 126 |
| Answers to Correspondents | 126 |

